# Synthesis, Characterization, and Photonic Efficiency of Novel Photocatalytic Niobium Oxide Materials

**DOI:** 10.1002/gch2.201700066

**Published:** 2017-11-02

**Authors:** Lidiane A. Morais, Cristina Adán, Antonio S. Araujo, Ana P. M. A. Guedes, Javier Marugán

**Affiliations:** ^1^ Department of Chemical and Environmental Technology ESCET Universidad Rey Juan Carlos C/Tulipán s/n 28933 Móstoles Madrid Spain; ^2^ Institute of Chemistry Universidade Federal do Rio Grande do Norte, Av. Senador Salgado Filho, n° 3000 Natal 59.078‐970 RN‐Brazil

**Keywords:** methanol, niobium oxides, photocatalysis, photonic efficiency, sodium niobates

## Abstract

The application of niobium oxides as photocatalytic materials for the removal of contaminants is scarcely reported in the literature. This work reports the methodology to synthesize four different mesoporous niobium oxide materials and the correlation between the physicochemical properties and the photocatalytic activity. X‐ray diffraction, UV–vis diffuse reflectance spectra (DRS), transmission electron microscopy, and nitrogen adsorption techniques are used to characterize the structure and composition of the obtained materials. The photocatalytic oxidation of methanol is used as reaction test to assess the photocatalytic activities and photonic efficiencies of the materials as a function of the catalyst concentration. Nb_2_O_5_ materials display lower reaction rates, which can be attributed to the relatively high average particle size. By contrast, NaNbO_3_ materials show higher activity, especially for high catalyst loading. No significant differences in absorption and scattering of light are observed among the materials, indicating that the higher photonic efficiency of NaNbO_3_ should be the result of a lower charge recombination derived from its microstructure, sodium composition, low particle size, and high specific surface area of these materials.

## 1 Introduction

The current levels of removal of water pollutants, achievable through the use of conventional water treatment technologies, are often not fully satisfactory. This fact makes the use of efficient processes for the removal and the degradation of organic contaminants necessary. Advanced oxidation processes are presented as interesting alternatives for the oxidation of aqueous hazardous organic pollutants refractory to conventional biological processes. These processes involve the generation of highly reactive hydroxyl radical (•OH) species. Among them, heterogeneous photocatalysis has attracted much attention due to its consideration as a green technology for application in water and air purification. Photocatalytic materials are wide‐band‐gap semiconductors activated by natural or artificial light to generate electron–hole pairs that eventually lead to the formation of different reactive oxygen species, including hydroxyl radicals, responsible for the degradation of the organic pollutants.^1^, ^2^ Titanium dioxide (TiO_2_) has been widely described as the most active photocatalyst that meets the criteria of high stability under different reaction conditions, resistance to photocorrosion, and high photoactivity. However, other materials with similar properties such as Fe_2_O_3_, CdS, SnO_2_, ZrO_2_, and ZnO, have demonstrated to achieve good photocatalytic activity for organic matter decontamination.^3^, ^4^ Among all these photocatalytic materials which exhibit similar properties than titanium oxide, niobium oxide‐based semiconductors have been scarcely studied. The combination of niobium with oxygen mainly exists in the form of stoichiometric oxides such as NbO, Nb_2_O_3_, NbO_2_, and Nb_2_O_5_, the latter being the most well‐known material.^5^ Nb_2_O_5_ presents strong surface acidity and stability in an aqueous medium and present low toxicity. Its band‐gap energy is of 3.4 eV,^5^, ^6^ a value close to that of TiO_2_ (about 3.2 eV^7^) and is, therefore, suitable for use as a photocatalyst under UV light. The estimated abundance of niobium in crustal Earth is of 20 mg kg^−1^, Brazil being the principal niobium producing country with about 60% of the total world production.^6^

The synthesis of niobium oxides and its compounds can be carried out by many different methods, including those leading to mesoporous transition metal oxides of high surface areas.^8^ The synthesis of mesoporous Nb_2_O_5_ materials is based on the use of poly(alkylene oxide) block copolymers, such as Pluronic 123, as structure‐directing agent.^6^, ^9^ The literature shows reports on niobium materials with photocatalytic properties, such as the niobium‐based perovskites KNbO_3_ or NaNbO_3_,^10^, ^11^ and customized morphologies, such as Nb_2_O_5_ nanopillars, nanobelts, and nanorods.^5^, ^12^, ^13^, ^14^ Increase in the sensitivity of Nb_2_O_5_ materials to visible light has also been reported by introducing metal and nonmetal dopants as well as modification with small band‐gap semiconductors to form heterojunctions.^8^, ^12^, ^14^, ^15^, ^16^

The photocatalytic properties of Nb_2_O_5_ and NaNbO_3_ for the degradation of organic pollutants in water have been previously reported.^6^, ^11^, ^17^, ^18^, ^19^ NaNbO_3_ is well‐known for exhibiting a rich polymorphism based on the perovskite structure that possesses attractive physical properties, including being nontoxic and highly stable; therefore, it is currently attracting much interest for its photocatalytic properties.^11^ NaNbO_3_ structures can be synthesized via the solid‐state reaction of alkali metal carbonates and Nb_2_O_5_ or by a surfactant‐assisted hydrothermal method as in Nb_2_O_5_ materials. Despite its many polymorphic structures, the orthorhombic phase of NaNbO_3_ has been the most studied in the photocatalytic process.^11^

This work constitutes a comprehensive and comparative study on the synthesis, characterization and evaluation of the photocatalytic activity of different niobium oxide materials, including Nb_2_O_5_, N‐doped Nb_2_O_5_, NaNbO_3_, and templated NaNbO_3_. The report focuses on the influence of the synthesis route on the physicochemical properties of the synthesized materials and their correlation with the photocatalytic activity, including the evaluation of the photonic efficiency as a function of the catalyst loading.

## 2 Results and Discussion

### 2.1 Characterization of the Niobium Photocatalysts

**Figure 1:**
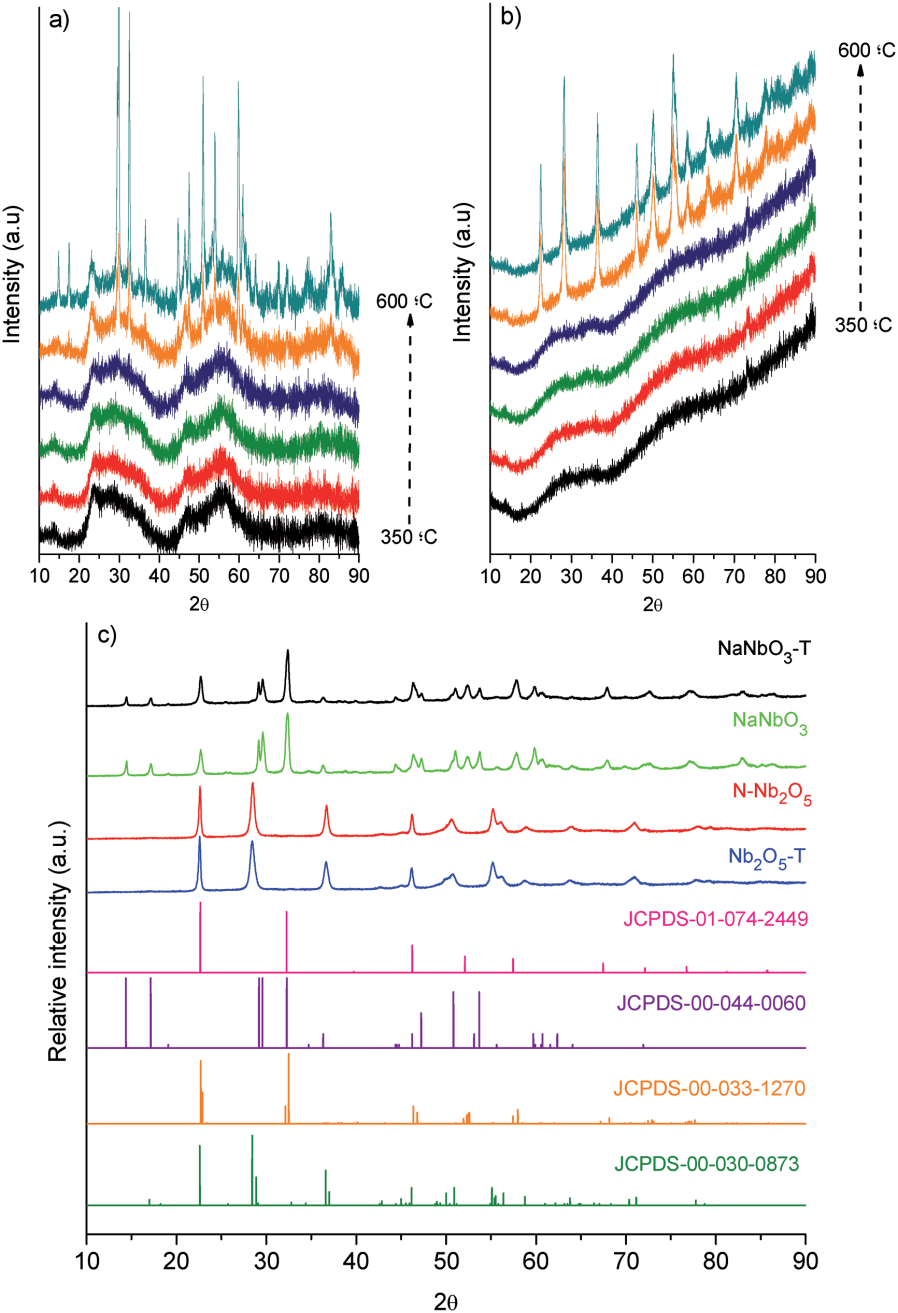
XRD patterns of niobium oxide materials at different calcination temperatures of 350, 400, 450, 500, 550, and 600 °C: a) NaNbO_3_ and b) N‐Nb_2_O_5_ and c) X‐ray powder diffraction patterns of the niobium oxide nanoparticles synthesized for different routes calcined at 600 °C. JCPDS standards indexed correspond to monoclinic NaNbO_3_ (01‐074‐2449 and 00‐044‐0060), orthorhombic NaNbO_3_ (00‐033‐1270) and orthorhombic Nb_2_O_5_ (00‐030‐0873).

X‐ray diffraction patterns of the synthesized niobium oxides are shown in **Figure** **1**. A preliminary study at different calcination temperatures (showed in Figure 1a,b) demonstrate that after heating at 500 °C the samples remain amorphous. Only on reaching temperatures higher than 550 °C can the main signals of different niobium crystal phases be detected. In Figure 1c the niobium oxides synthesized for different routes and calcined at 600 °C are shown. Interestingly, the materials prepared without using NaOH present a well‐defined crystalline structure which are indexed to orthorhombic (Pbma) Nb_2_O_5_ structure (according to the JCPDS file no. 00‐030‐0873). Meanwhile, the materials prepared in NaOH solutions show the formation of a NaNbO_3_ structure, displaying a mixture of crystalline phases. The coexistence of sodium niobate phases can be indexed to monoclinic and orthorhombic structures, confirmed with the standards diffraction data (JCPDS file no. 01‐074‐2449, JCPDS file no. 00‐044‐0060 for monoclinic and JCPDS file no. 00‐033‐1270 for orthorhombic), according to other works.^20^, ^21^

**Table** **1** summarized the main physicochemical properties of the niobium oxides. Average crystallite sizes of Nb_2_O_5_ and NaNbO_3_ materials were calculated by the main diffraction signals observed at 2θ values of 28.40 and 22.64, respectively. All the catalysts display relatively large crystals although those of NaNbO_3_ materials are slightly smaller.

**Table 1:** Main physicochemical parameters of niobium oxide samples

Catalysts	Average crystallite size [nm]	*S*_BET_ [m^2^ g^−1^]	Micropore area [m^2^ g^−1^]	Cumulative pore volume [cm^3^ g^−1^]	O/Nb atomic ratioa)	Na/Nb atomic ratioa)	N/Nb atomic ratioa)	Specific extinction coefficient β* [cm^2^ g^−1^]
NaNbO_3_	28	26.8	13.5	0.131	1.25	0.11	–	0.147
NaNbO_3_‐T	77	13.0	7.4	0.012	1.25	2.5	–	0.152
Nb_2_O_5_‐T	62	19.0	9.8	0.026	1.25	–	–	0.160
N‐Nb_2_O_5_	36	14.9	7.7	0.020	1.6	–	0.08	0.164

^a)^ Atomic ratio determined by EDX.

**Figure 2:**
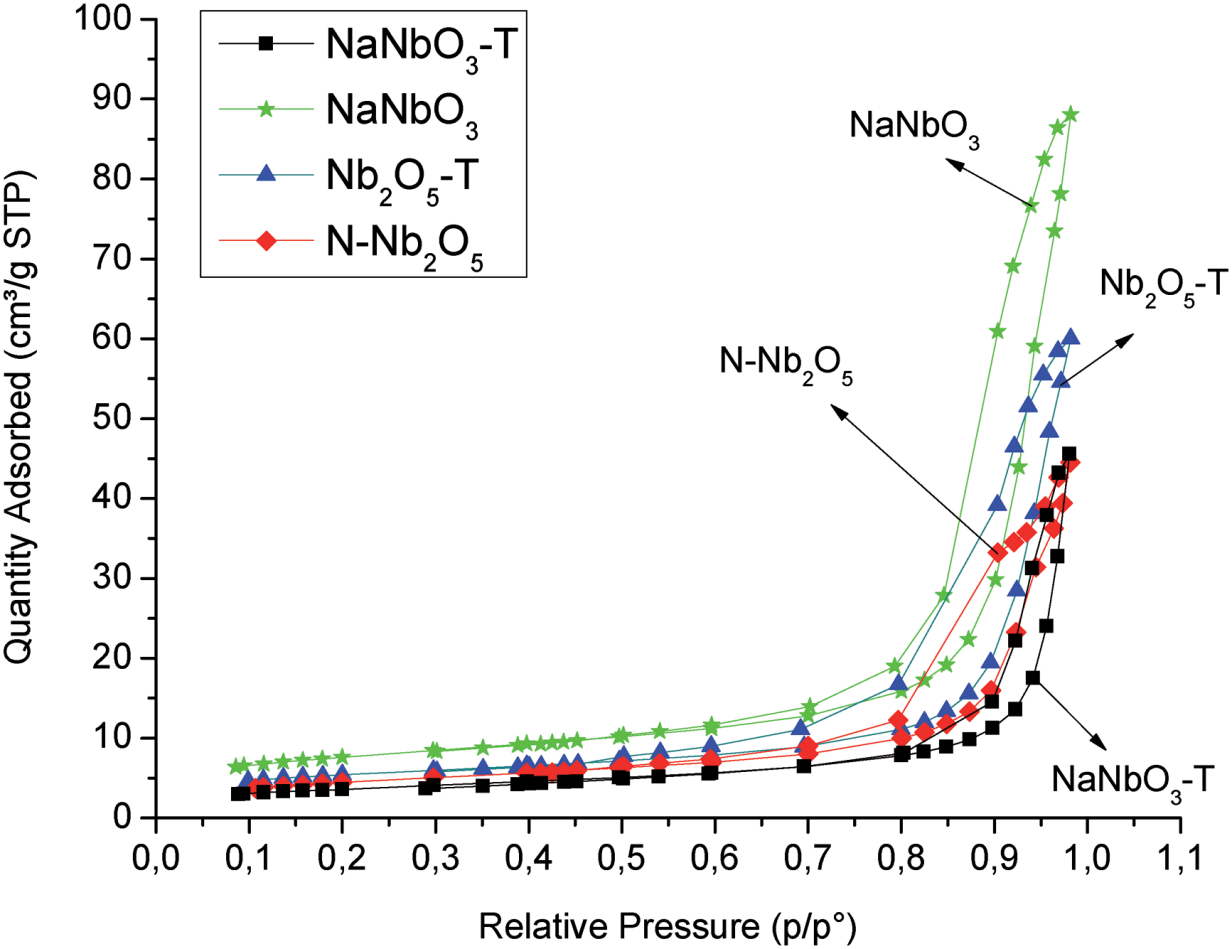
Nitrogen adsorption–desorption isotherms for all studied niobium oxide nanoparticulate photocatalysts.

**Figure** **2** shows the nitrogen adsorption/desorption isotherms of the studied materials. The NaNbO_3_ material shows greater adsorbed gas volume, as a result of the larger micropore volume and a larger surface area in comparison with the other niobium oxides^22^ (data summarized in Table 1). The calculated specific surface areas of niobium oxides are in agreement with previous studies,^6^ being relatively low due to the high calcination temperature required to induce the formation of crystalline phases. As clearly shown in Table 1, the surface area of the NaNbO_3_ material (26.8 m^2^ g^−1^) is slightly higher suggesting that the smaller crystallites lead to the formation of a microporous structure.

**Figure 3:**
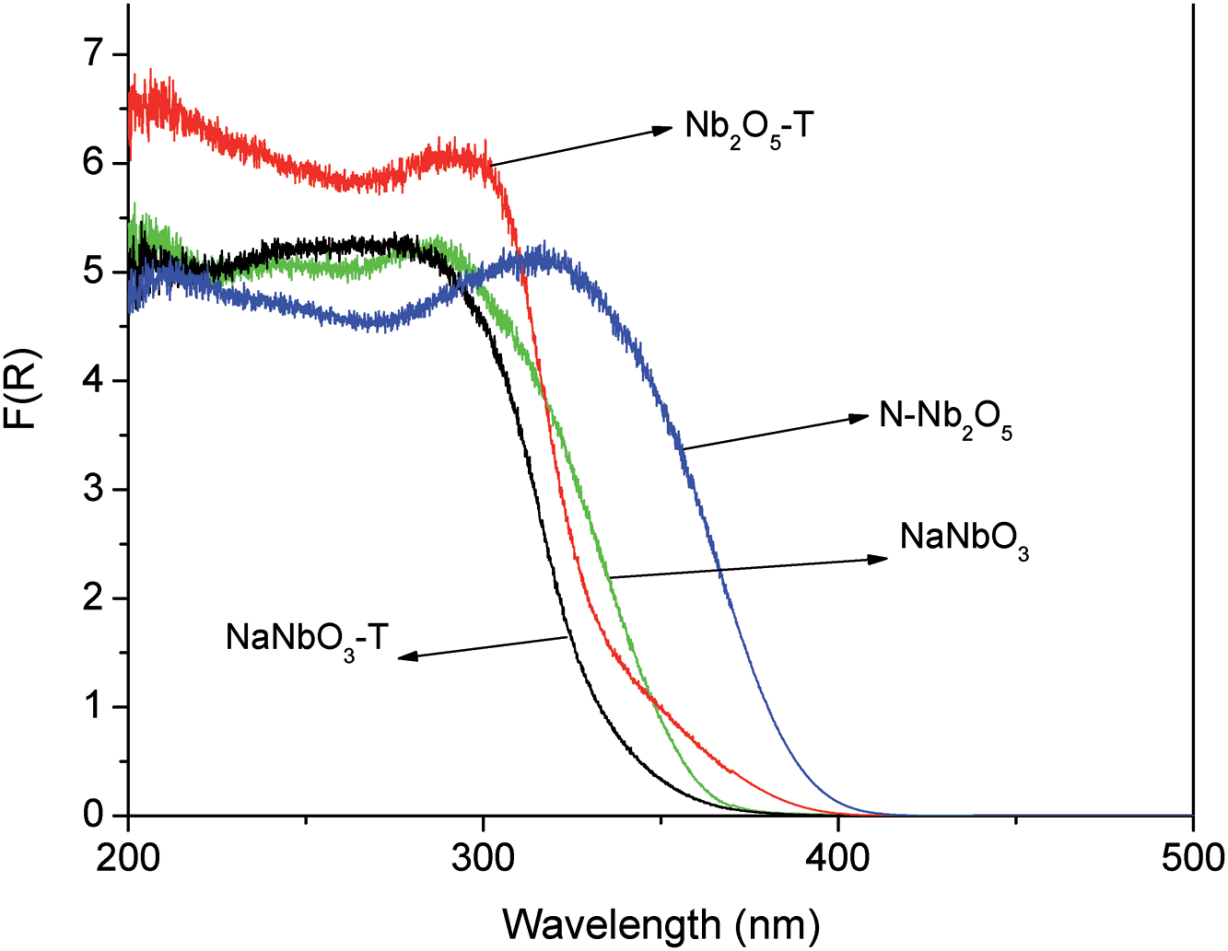
UV–vis diffuse reflectance spectra of indicated niobium oxide samples.

**Figure** **3** shows the UV–vis diffuse reflectance spectra of the synthesized niobium oxides. The absorption spectra are represented in terms of the Kubelka–Munk function. All the materials show absorption in the ultraviolet range between 200 and 400 nm attributed to the intrinsic band gap absorption of the semiconductor crystals. The studied Nb_2_O_5_ materials display less energetic absorption edges than the NaNbO_3_ samples. In addition, the N‐Nb_2_O_5_ sample doped with nitrogen present a shift toward the visible spectrum in the absorption band in comparison with the Nb_2_O_5_‐T sample, since nitrogen doping caused a reduction in the apparent optical band gap energy, positively affecting the visible‐light‐induced photocatalysis.^12^, ^15^

**Figure 4:**
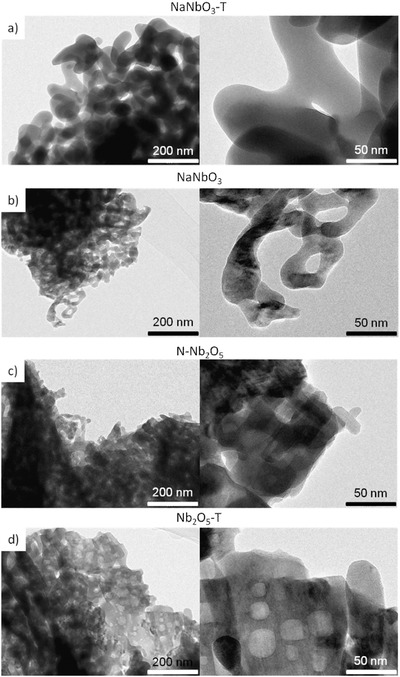
TEM images at different magnifications of the niobium oxide nanoparticles: a) NaNbO_3_, b) NaNbO_3_‐T, c) N‐Nb_2_O_5_, and d) Nb_2_O_5_‐T.

Scanning electron microscopy (SEM) was used to analyze the particle morphology. SEM images (Figure S1 in the Supporting Information) show big particles and particle aggregates of sizes higher than 20 µm constituted of small crystals. Transmission electron microscopy (TEM) images of the niobium photocatalysts are shown in **Figure** **4** (additional magnification images in Figure S2 in the Supporting Information). It is clearly observed that the morphology of the material synthesized without NaOH is quite different from those synthesized with NaOH. According to the TEM images, the sodium niobates and niobium oxide materials differ in particle shape, morphology, and porous structure, depending on the material and the sodium concentration. It should be noticed that pure Nb_2_O_5_ samples consist in agglomerates of crystallites with a denser structure formed by small particle grains and the wormhole‐like mesoporous structure while the NaNbO_3_ materials appear to be formed by longer interlocking crystals that lead to a structure with big empty holes. The shape of the crystals makes the measurements and the estimation of quantitative particle sizes difficult since the edges of the crystal are not well defined.

Regarding composition, Table 1 summarizes the atomic ratios (estimated from the energy‐dispersive X‐ray (EDX) analyses) of the niobium oxides and sodium niobates. In all cases, the O/Nb atomic ratio in the materials is lower than the theoretical value demonstrating the existence of oxygen vacancies in the structures. On the other hand, the nitrogen amount introduced in the N‐Nb_2_O_5_ material is relatively low (2.8 at%), indicating certain difficulties in the incorporation of nitrogen into the niobium oxide by this synthesis method. However, as observed in Table 1, the amount of Na recorded with EDX is much higher in the NaNbO_3_‐T sample (almost twice the amount of niobium) than in the NaNbO_3_. This observation could be explained by assuming that during the synthesis of this material the templating agent is helping to the introduction of sodium in the NaNbO_3_‐T structure. In any case, both NaNbO_3_ samples can be considered far from stoichiometric materials.

### 2.2 Photocatalytic Activity of Niobium Samples for Methanol Oxidation

**Figure 5:**
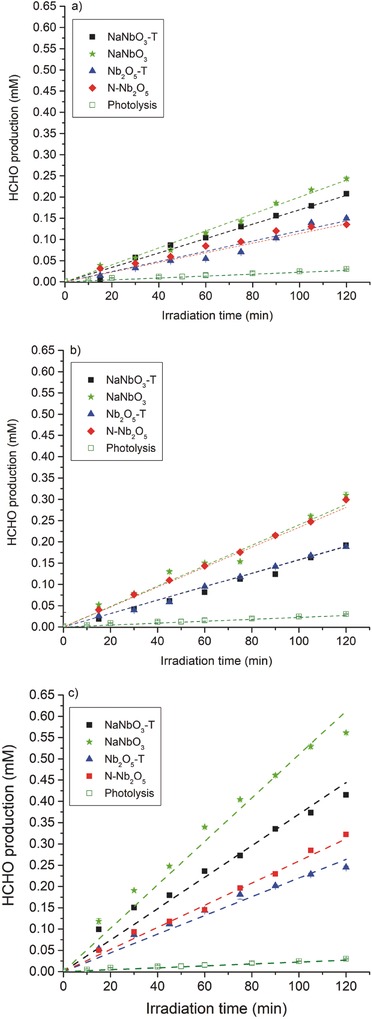
Formaldehyde formation from the photocatalytic oxidation of methanol with the studied niobium oxide materials with different catalyst loading of a) 1 g L^−1^, b) 2 g L^−1^, c) 4 L^−1^.

**Figure** **5** shows the formaldehyde concentration profiles obtained during the photocatalytic oxidation of methanol with different concentrations of the studied materials in comparison with the reference photolytic reaction. This photocatalytic reaction leads to linear formaldehyde concentration profiles that can be adjusted to a zero‐order kinetic constant (*k*) directly equivalent to the reaction rate (*r*_o_).^23^

As expected, the photocatalytic activity increases for higher catalyst loadings due to the increase in the absorption of photons. The concentration of catalysts strongly depends on the optical properties of the materials (and of course on the incident light power), being in the order of 0.1–0.2 g L^−1^ for TiO_2_ P25 (exact value depends on the geometry of the reactor and the available light). In the case of the studied niobium oxide materials, the increase in activity is still observed even for catalyst concentration of 4 g L^−1^, suggesting that the material suspensions have relatively low specific absorption coefficients. Determination of niobium concentration dissolved in the solution at the end of the reaction by ICP/AES was found to be negligible. Moreover, the recovery of the catalysts and further reuse in subsequent reactions leads to comparable activity, confirming the stability of the developed materials.

**Figure 6:**
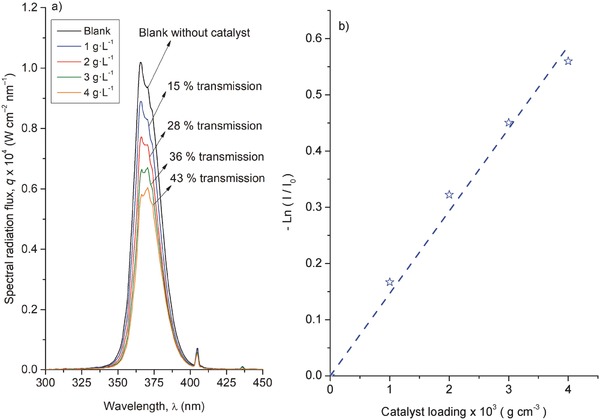
a) Example of spectral radiation fluxes transmitted through the photoreactor for increasing concentrations and b) Attenuation of transmitted radiation versus catalysts loading of NaNbO_3_ material.

An example of spectral radiation fluxes in the outer wall of the reactor from the UV‐A lamp with increasing suspensions of the NaNbO_3_ catalyst is shown in **Figure** **6**a (radiation measurements with all the studied materials are shown in Figure S3 in the Supporting Information). As expected, an increase in catalyst concentration leads to a reduction in the outgoing light flux (also resulting in an increase in the methanol degradation rate). Figure 6b shows the plot of the radiation extinction versus catalyst loading. A clear linear trend can be observed, being the slope of the specific extinction coefficient (β* ^)^ of the material (optical path of the reactor is 1 cm). Values of β* of the different materials are shown in Table 1, not showing significant differences.

On the other side, the efficiency of the photocatalytic process can be represented by the effectiveness in the use of radiation by means of the photonic efficiency that has been determined as the ratio between the amount of product formed per unit of time (mol s^−1^) and the amount of photons entering the reactor per unit of time (Einstein s^−1^).

**Figure 7:**
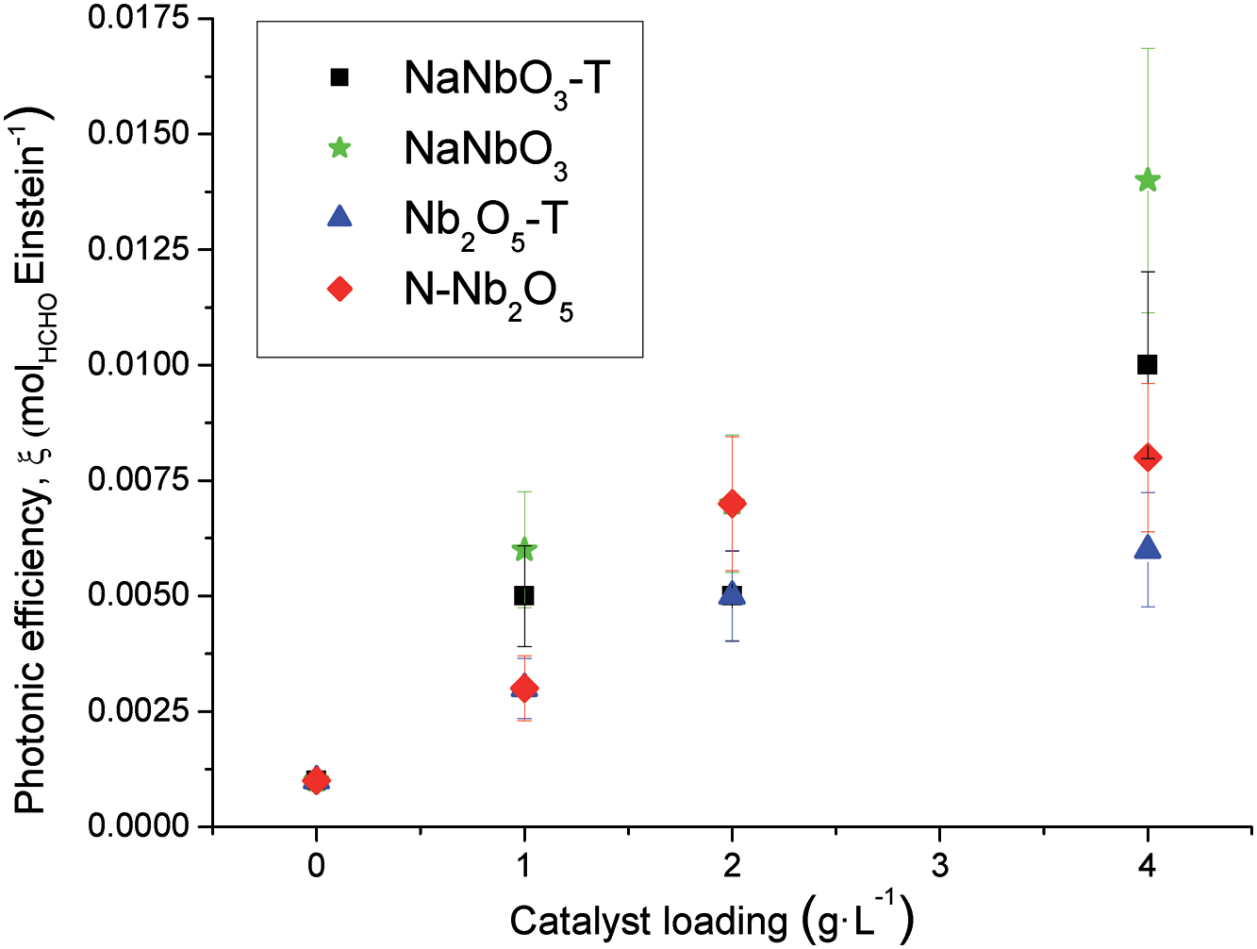
Photonic efficiencies of formaldehyde formation during the photocatalytic oxidation of 0.1 m solutions of methanol.

**Figure** **7** shows the calculated photonic efficiencies for the photocatalytic oxidation of methanol at increasing catalyst loading. As shown in Figure 7, the photonic efficiency of NaNbO_3_ materials increases with increasing catalyst loading and becomes even larger at a loading higher than 2 g L^−1^, being much higher than the values of Nb_2_O_5_ materials.

The Nb_2_O_5_ materials lead to different photonic efficiencies that do not correlate with the specific extinction coefficient values. This fact suggests that there are significant differences in their photochemical efficiencies (in terms of photons to product molecules) that mostly depend on the physicochemical properties of the materials. In all the systems the photons reach the catalyst that is able to absorb a part of the irradiation that will generate the electron–hole charges. At the same time, the generated charges would be partially transferred to water molecules in the semiconductor–electrolyte interface for the production of hydroxyl radicals. The Nb_2_O_5_ samples could be able to absorb a higher amount of radiation, but the reaction rate is lower than expected probably due to a higher recombination of charges. On the contrary the NaNbO_3_ structures show good photocatalytic activity, especially at a high catalyst concentration. In particular, the NaNbO_3_ catalyst is clearly the most active photocatalyst for HCHO formation from CH_3_OH under these conditions. The comparatively low activity of Nb_2_O_5_ materials can be attributed to a particle size effect. It is known that a decrease in particle size leads to the reduction of volume recombination because the migration time of photogenerated charge carriers to the surface is proportional to the square of the particle size.^24^ Obviously, although the NaNbO_3_ catalyst presents a smaller crystal particle size and larger surface area than the other based niobium oxides, the activity of these photocatalysts is not only controlled by particle size. Additional factors such as microstructure, sodium impurities, agglomeration and specific surface properties have to be taken into account. In any case, the photon efficiency of these NaNbO_3_ materials is higher than niobium oxides synthesized in this work which makes them potentially useful in the photocatalytic degradation of organic compounds.

## 3 Conclusions

A series of niobium‐based photocatalytic materials with mesoporous structure were synthetized by using a refluxing method with and without the use of NaOH in different preparation conditions. Increased calcination temperatures exhibited a strong influence on the structure formation and crystallinity of mesoporous materials. Up to 550 °C, NaNbO_3_ and Nb_2_O_5_ systems, with different structures have been obtained. The synthesis conditions have determined the formation of two different photocatalytic materials. The orthorhombic Nb_2_O_5_ pure structure has been obtained without using NaOH while the NaNbO_3_ perovskite structure was prepared in NaOH solutions. These materials show the coexistence of various monoclinic and orthorhombic crystalline phases. Methanol degradation results show that photocatalytic activity is strongly dependent on catalyst concentration due to the low absorption of these materials. No significant differences in the specific extinction coefficients of NaNbO_3_ and Nb_2_O_5_ materials were observed, indicating that they present a similar interaction with radiation. However, NaNbO_3_ materials show higher reaction rates than those of niobium oxides, indicating that the higher photonic efficiencies are not based on higher radiation absorption but on higher charge transfer rate derived from the differences in the structural, surface and chemical properties of these materials. The reduction of crystalline particle size combined a higher surface and microporous area in comparison with the other studied materials make this catalyst more promising for the photocatalytic degradation of organic pollutants in water.

## 4 Experimental Section

**Figure 8:**
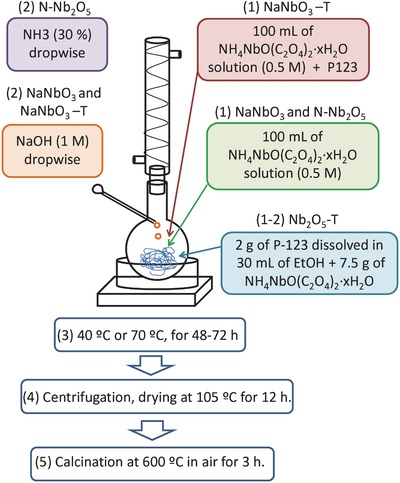
Schematic representation of experimental procedure used for the preparation of the niobium oxide nanoparticulate photocatalysts. Numbers (1) and (2) represent the steps characteristics of the preparation of different materials.

*Synthesis of Niobium Oxide Materials*: The niobium oxide materials were prepared by a refluxing method with different modifications using as precursor ammonium niobium oxalate supplied by the Companhia Brasileira de Metalurgia e Mineração (CBMM). For the synthesis of the NaNbO_3_, 100 mL of a 0.5 m solution of ammonium niobium oxalate (NH_4_NbO(C_2_O_4_)_2_·xH_2_O, Sigma–Aldrich) was placed in a round bottom flask. Afterward, 50 mL of NaOH (1 m) were added dropwise to the flask until pH 6 to ensure the complete precipitation of niobium oxide particles. The mix was refluxed at a temperature of 70 °C under agitation during 72 h. Then, the solid phase was separated by centrifugation, washed repeatedly with abundant Milli‐Q water (18.2 MΩ cm) until pH 7 and dried at 105 °C for 12 h. The NaNbO_3_‐T (T = Template) and N‐Nb_2_O_5_ materials were prepared in the same way with some exceptions: i) in the case of NaNbO_3_‐T, 10 g of template Pluronic P123 (polyethylene glycol ‐ polypropylene glycol ‐ polyethylene glycol (PEG‐PPG‐PEG) symmetric triblock copolymer, Sigma–Aldrich) was added to the ammonium niobium oxalate solution and the mix was refluxed at 40 °C under agitation during 72 h, ii) for the doping of nitrogen N‐Nb_2_O_5_ material, a NH_3_ solution (30% v/v) was used instead of NaOH. For the synthesis of Nb_2_O_5_‐T, 7.5 g of an ammonium niobium oxalate was dissolved in 30 mL of ethanol and 2 g of Pluronic P123 was added to this mix and placed in a round bottom flask at 40 °C during 48 h, adapted from the work of Chen et al.^9^ The resulting gel was centrifuged and dried at 105 °C for 12 h. All the samples were finally calcined at 600 °C during 3 h to induce the formation of the crystalline phases required for the semiconductor photocatalysis activity. **Figure** **8** shows the schematic representation of the different steps followed for the synthesis of the niobium oxides.

*Materials Characterization*: X‐ray powder diffraction (XRD) patterns were obtained using a PhillipsX‐Pert‐MPD diffractometer with Cu Kα radiation equipped with an XCell detector. Data were collected from 14° to 90°. The study of the calcination temperature of the Nb_2_O_5_ was estimated in a temperature chamber of the diffractometer with a heating ramp of 10 °C min^−1^ up to 100 °C and 2 °C min^−1^ up to 600 °C. Diffractograms were made at the temperatures of 350, 400, 450, 500, 550, and 600 °C. Each diffractograms were performed with a scanning speed of 0.01° s^−1^ and with an accumulation time of 2 s per point. The structural identification was performed by using the JCPDS cards.^25^ Average crystallite sizes were calculated applying the Scherrer's equation.^26^

Specific surface areas were obtained by the Brunauer–Emmett–Teller (BET) method applied to the nitrogen‐adsorption isotherms registered with liquid‐nitrogen temperature at 77 K using a Micromeritics Tristar 3000 instrument.

UV–vis diffuse reflectance absorption spectra of the photocatalysts were recorded with a Varian Cary 500 Scan UV–vis–NIR apparatus in the 200–600 nm regions. The analysis of the band gap transitions from the samples was made using well‐known equations available in the literature.^27^

SEM images were taken with a Nova Nano SEM230 (FEG‐SEM) microscope working with acceleration voltages between 2 y 10 kV.

High Resolution Transmission electron microscopy microphotographs coupled to EDX microanalysis were done on a Philips Tecnal‐20 electron transmission microscope.

*Photocatalytic Tests*: The experimental setup for the photocatalytic oxidation methanol consisted of an annular photoreactor 15 cm in length, with inner and outer diameters of 3 and 5 cm, respectively, working in recirculation with a reservoir tank of 1 L. The catalysts were illuminated from the backside by a Philips TL 6W black light lamp (spectral emission centered at 370 nm) placed inside the inner tube of the reactor. More details of the reactor can be found elsewhere.^28^ The experiments have been carried out using catalyst concentrations of 1, 2, and 4 g L^−1^.

All reaction experiments lasted for 2 h and have been carried out in deionized water at natural pH with an initial concentration of methanol of 0.1 m. The photocatalytic activity was assessed by the reaction rate of formaldehyde formation, quantitative oxidation product when methanol is in excess.^29^ The concentration of formaldehyde was followed by colorimetric determination through the transformation to 1,4‐dihydro‐3,5 lutidine diacetyl, a yellowish compound that can be espectrophotometrically measured at 412 nm.^30^

The spectral transmission of the suspensions of these materials has been measured for all the catalyst concentrations with a BlueWave spectroradiometer (StellarNet Inc.) with a UV–vis–NIR cosine receptor calibrated in the range of 300–1100 nm.

## Conflict of Interest

The authors declare no conflict of interest.

## Supporting information

Supplementary
