# Supplementary material for: Synthesis, Characterization, and Photonic Efficiency of Novel Photocatalytic Niobium Oxide Materials
Source: Glob Chall. 2017 Nov 2;1(9):1700066. doi: 10.1002/gch2.201700066 (PMC6607167; doi:10.1002/gch2.201700066)
Supplement: Supplementary file 1 — Supplementary [file GCH2-1-1700066-s001.pdf]

Copyright WILEY-VCH Verlag GmbH & Co. KGaA, 69469 Weinheim, Germany, 2017.

Global Challenges
Open Access

Supporting Information

for *Global Challenges*, DOI: 10.1002/gch2.201700066

Synthesis, Characterization, and Photonic Efficiency of Novel Photocatalytic Niobium Oxide Materials

*Lidiane A. Morais, Cristina Adán, Antonio S. Araujo, Ana P. M. A. Guedes, and Javier Marugán**

SUPPLEMENTARY DATA

Figure 1S. SEM micrographs of the niobium oxide nanoparticles: a) $NaNbO_3$, b) $NaNbO_3$-T c) N-Nb_2O_5 and d) Nb_2O_5-T.

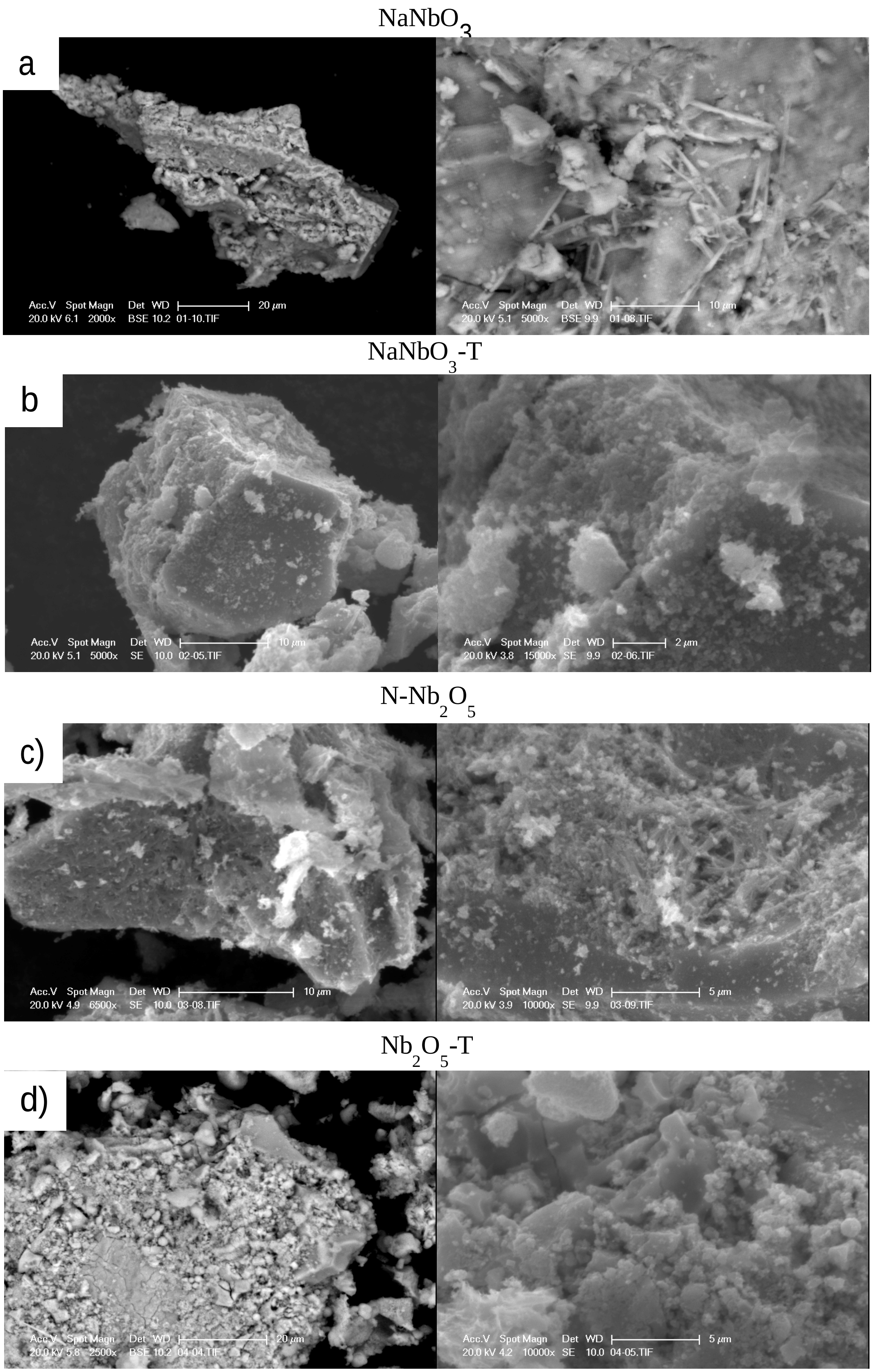
NaNbO$_3$
a
Acc.V Spot Magn Det WD 20 μm
20.0 kV 6.1 2000x BSE 10.2 01-10.TIF
Acc.V Spot Magn Det WD 10 μm
20.0 kV 5.1 5000x BSE 9.9 01-08.TIF
NaNbO$_3$-T
b
Acc.V Spot Magn Det WD 10 μm
20.0 kV 5.1 5000x SE 10.0 02-05.TIF
Acc.V Spot Magn Det WD 2 μm
20.0 kV 3.8 15000x SE 9.9 02-06.TIF
N-Nb$_2$O$_5$
c)
Acc.V Spot Magn Det WD 10 μm
20.0 kV 4.9 6500x SE 10.0 03-08.TIF
Acc.V Spot Magn Det WD 5 μm
20.0 kV 3.9 10000x SE 9.9 03-09.TIF
Nb$_2$O$_5$-T
d)
Acc.V Spot Magn Det WD 20 μm
20.0 kV 5.8 2500x BSE 10.2 04-04.TIF
Acc.V Spot Magn Det WD 5 μm
20.0 kV 4.2 10000x SE 10.0 04-05.TIF

Figure 2S. Higher magnification Transmission Electron Microscopy (TEM) images from: a) $NaNbO_3$, b) $NaNbO_3$-T c) N-Nb_2O_5 and d) Nb_2O_5-T.

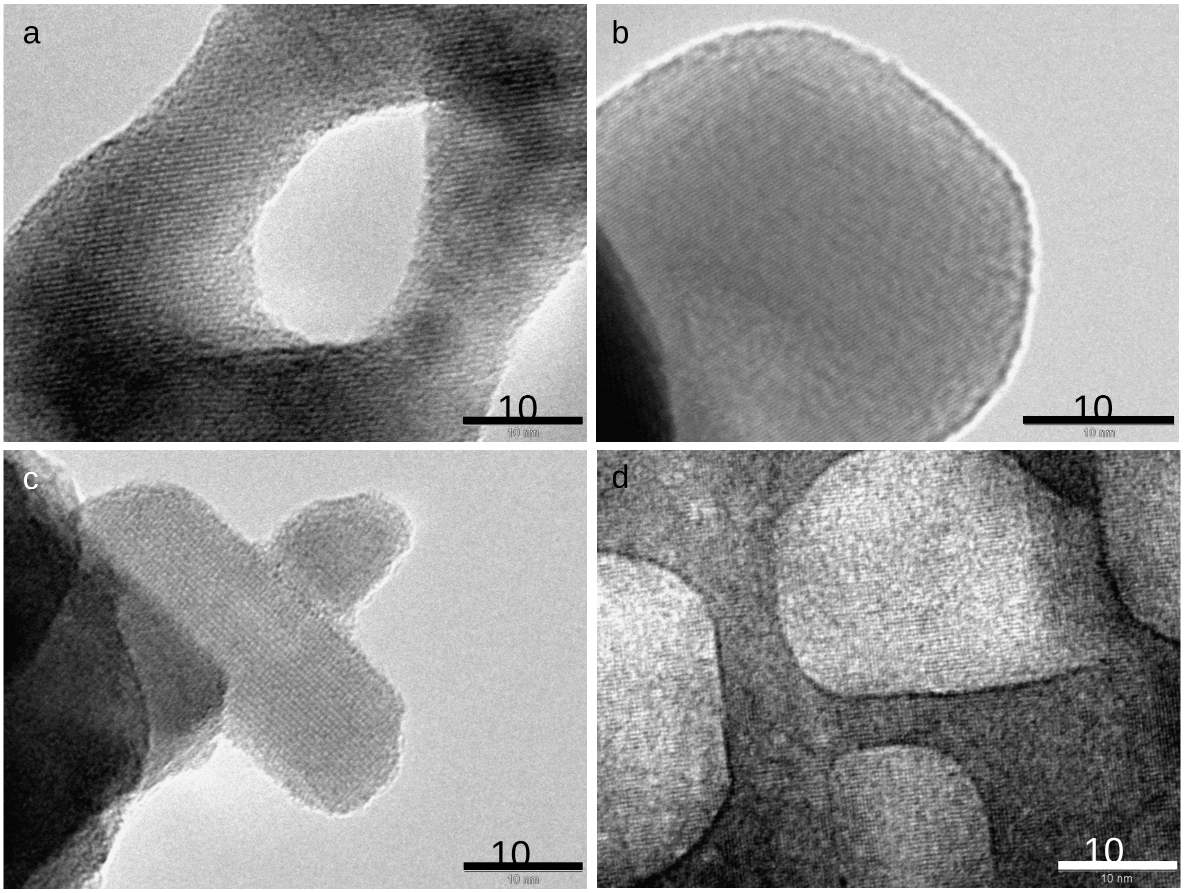

Figure 3S. Spectral radiation fluxes from the UVA lamp entering the reactor volume after crossing the inner glass tube and catalyst with increasing concentrations of 1, 2, 3 and 4 $g \cdot L^{-1}$ of niobium oxide samples: a) $NaNbO_3$, b) $NaNbO_3$-T c) N-Nb_2O_5 and d) Nb_2O_5-T.

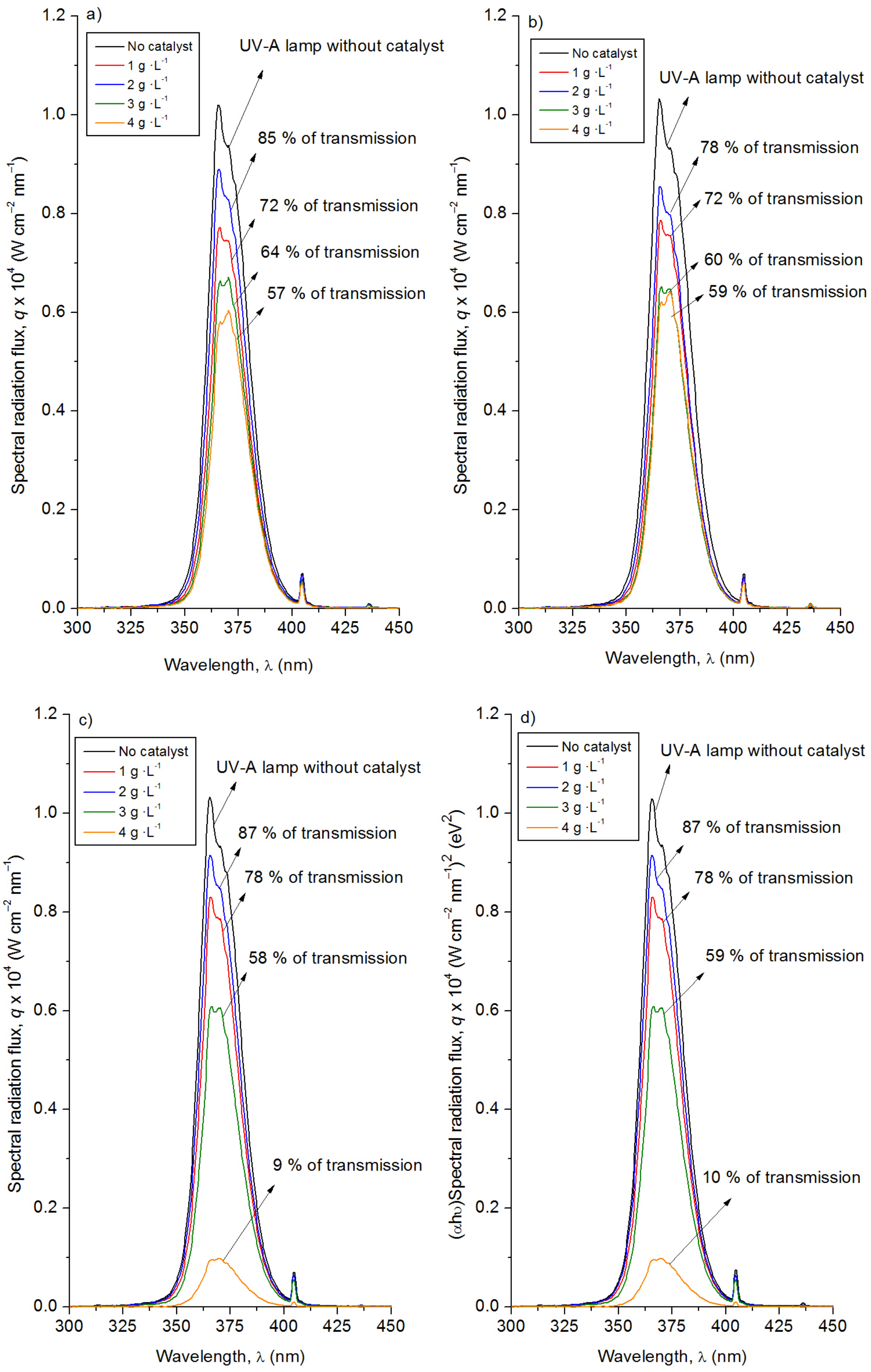
a)
No catalyst
1 g ·L⁻¹
2 g ·L⁻¹
3 g ·L⁻¹
4 g ·L⁻¹
UV-A lamp without catalyst
85 % of transmission
72 % of transmission
64 % of transmission
57 % of transmission
Spectral radiation flux, q x 10⁴ (W cm⁻² nm⁻¹)
Wavelength, λ (nm)
b)
No catalyst
1 g ·L⁻¹
2 g ·L⁻¹
3 g ·L⁻¹
4 g ·L⁻¹
UV-A lamp without catalyst
78 % of transmission
72 % of transmission
60 % of transmission
59 % of transmission
Spectral radiation flux, q x 10⁴ (W cm⁻² nm⁻¹)
Wavelength, λ (nm)
c)
No catalyst
1 g ·L⁻¹
2 g ·L⁻¹
3 g ·L⁻¹
4 g ·L⁻¹
UV-A lamp without catalyst
87 % of transmission
78 % of transmission
58 % of transmission
9 % of transmission
Spectral radiation flux, q x 10⁴ (W cm⁻² nm⁻¹)
Wavelength, λ (nm)
d)
No catalyst
1 g ·L⁻¹
2 g ·L⁻¹
3 g ·L⁻¹
4 g ·L⁻¹
UV-A lamp without catalyst
87 % of transmission
78 % of transmission
59 % of transmission
10 % of transmission
(αhυ)Spectral radiation flux, q x 10⁴ (W cm⁻² nm⁻¹)² (eV²)
Wavelength, λ (nm)
